# Mild Inflammatory Profile without Gliosis in the c-Rel Deficient Mouse Modeling a Late-Onset Parkinsonism

**DOI:** 10.3389/fnagi.2017.00229

**Published:** 2017-07-19

**Authors:** Vanessa Porrini, Mariana Mota, Edoardo Parrella, Arianna Bellucci, Marina Benarese, Lara Faggi, Paolo Tonin, Pier F. Spano, Marina Pizzi

**Affiliations:** ^1^Laboratory of Pharmacology, Department of Molecular and Translational Medicine, University of Brescia Brescia, Italy; ^2^IRCCS, San Camillo Hospital Venice, Italy

**Keywords:** c-Rel, neuroinflammation, microglia, Parkinson’s disease, astrocytes, substantia nigra

## Abstract

The impact of neuroinflammation and microglial activation to Parkinson’s disease (PD) progression is still debated. Post-mortem analysis of PD brains has shown that neuroinflammation and microgliosis are key features of end-stage disease. However, microglia neuroimaging studies and evaluation of cerebrospinal fluid (CSF) cytokines in PD patients at earlier stages do not support the occurrence of a pronounced neuroinflammatory process. PD animal models recapitulating the motor and non-motor features of the disease, and the slow and progressive neuropathology, can be of great advantage in understanding whether and how neuroinflammation associates with the onset of symptoms and neuronal loss. We recently described that 18-month-old NF-κB/c-Rel deficient mice (c-rel^−/−^) develop a spontaneous late-onset PD-like phenotype encompassing L-DOPA-responsive motor impairment, nigrostriatal neuron degeneration, α-synuclein and iron accumulation. To assess whether inflammation and microglial activation accompany the onset and the progression of PD-like pathology, we investigated the expression of cytokines (*interleukin 1 beta (Il1b), interleukin 6 (Il6)*) and microglial/macrophage activation markers (*Fc gamma receptor III (Fcgr3), mannose receptor 1 (Mrc1), chitinase-like 3 (Ym1), arginase 1 (Arg 1), triggering receptor expressed on myeloid cells 2 (Trem2)*), together with microglial ionized calcium binding adapter molecule 1 (Iba1) and astrocyte glial fibrillary acidic protein (GFAP) immunolabeling, in the substantia nigra (SN) of c-rel^−/−^ mice, at premotor (4- and 13-month-old) and motor phases (18-month-old). By quantitative real-time RT-PCR we found increased M2c microglial/macrophage markers expression (*Mrc1* and *Arg1*) in 4-month-old c-rel^−/−^ mice. M2-type transcription dropped down in 13-month-old c-rel^−/−^ mice. At this age, the pro-inflammatory *Il1b*, but not *Il6* or the microglia-macrophage M1-polarization marker *Fcgr3*/CD16, increased when compared to wild-type (wt). Furthermore, no significant variation in the transcription of inflammatory and microglial/macrophage activation genes was present in 18-month-old c-rel^−/−^ mice, that display motor dysfunctions and dopaminergic neuronal loss. Immunofluorescence analysis of Iba1-positive cells in the SN revealed no sign of overt microglial activation in c-rel^−/−^ mice at all the time-points. MRC1-Iba1-positive cells were identified as non-parenchymal macrophages in 4-month-old c-rel^−/−^ mice. Finally, no sign of astrogliosis was detected in the SN of the diverse animal groups. In conclusion, this study supports the presence of a mild inflammatory profile without evident signs of gliosis in c-rel^−/−^ mice up to 18 months of age. It suggests that symptomatic PD-like phenotype can develop in the absence of concomitant severe inflammatory process.

## Introduction

Parkinson’s disease (PD) is the major neurodegenerative motor disorder worldwide, characterized by motor symptoms such as bradykinesia, resting tremor and rigidity. Key hallmarks of the brains of affected patients are the loss of dopaminergic neurons of substantia nigra (SN) pars compacta and Lewy bodies, intracellular inclusions mainly composed by aggregated α-synuclein (Poewe et al., 2017). Although the neuropathological features of the disease have been well described, the biological basis of neuronal death still needs to be clarified. Nonetheless, processes such as neuroinflammation and microglial activation have been proposed to contribute to the onset of PD (Poewe et al., 2017).

Microglia, the resident immune cells of the brain (Aguzzi et al., 2013), are essential for brain homeostasis in physiological conditions (Nimmerjahn et al., 2005; Neumann et al., 2009; Miyamoto et al., 2013). During brain injury, microglia become activated, which can be either detrimental or protective for neuronal cells in the central nervous system (CNS; Hayakawa et al., 2008; Brites and Vaz, 2014; Hu et al., 2015). The opposite role of microglia in brain diseases has been hypothesized to arise from the fact that these cells can adopt diverse activation states (Tang and Le, 2016). In particular, the classification of monocyte-derived macrophages into classically (M1) and alternatively activated (M2) was later applied to microglia (Gordon, 2003). While the M1 state has been associated with pro-inflammatory responses (Hu et al., 2015), the alternatively activated state M2 has been found to be related to healing and scavenging. In addition, three different M2 sub-classes have been described: M2a, M2b and M2c, that have been associated with tissue repair, regulation of inflammation and tissue remodeling, and debris and iron scavenging, respectively (David and Kroner, 2011).

The first evidence of increased microglial activation in post-mortem brains of PD patients was published by McGeer and collaborators almost 30 years ago (McGeer et al., 1988). Since then, microgliosis and inflammation have been thought to play a role in PD pathogenesis and/or progression (Long-Smith et al., 2009). On the other hand, the relevance of astrogliosis in PD pathology remains debated (Hirsch and Hunot, 2009), due to contrasting results of studies in PD patients (Knott et al., 1999; Mirza et al., 2000; Thannickal et al., 2007; Song et al., 2009; Tong et al., 2015). Indeed, post-mortem analysis of PD brains showed increased reactive microglia in the SN around the surviving dopaminergic neurons, as well as an augmented level of cytokines (McGeer et al., 1988; Mogi et al., 1994a; Banati et al., 1998; Imamura et al., 2003), indicating a clear involvement of microglia and inflammation at the end stage of the disease. In order to evaluate neuroinflammation during different stages of the disease, several studies have analyzed the content of cytokines in the cerebrospinal fluid (CSF) of patients affected by PD, which is considered a representative indicator of pathological brain states. Earlier studies found an increase in the levels of cytokines, such as interleukin 6 (IL-6), interleukin 1 beta (IL-1β), TNFα and TGF-β, in CSF from relatively small cohorts of PD patients (Mogi et al., 1994b, 1995, 1996; Blum-Degen et al., 1995; Vawter et al., 1996; Müller et al., 1998). On the other hand, more recent studies on larger patients’ cohorts showed no differences in the CSF levels of IL-6 and TNFα (Lindqvist et al., 2013), and fractalkine (Shi et al., 2011), in PD subjects compared to controls. Thus, while clear neuroinflammatory pathology has been established in the post-mortem brain of late-stage PD, it remains arguable the participation of inflammatory processes with microglial activation in the earlier and progressive stages of PD.

Animal models of PD could be valuable tools to investigate whether inflammation with microglial activation, and its diverse activation states, occurs along with SN degeneration and the onset of motor dysfunction. However, none of the available models fully recapitulates the pathological hallmarks and the slow progression of PD (Cebrián et al., 2015). Thus, the importance of these pathological features to the onset and worsening of the disease is still an open question.

We have previously shown that mice deficient for the NF-κB c-Rel nuclear factor (c-rel^−/−^ mice) develop a late-onset parkinsonism (Baiguera et al., 2012) characterized by significant impairment in spontaneous motor activity. They displayed an L-DOPA-reversible hypo-motility and gait-related deficits at 18 months, but not at younger ages (Baiguera et al., 2012). The motor deficits observed in aged c-rel^−/−^ mice were accompanied by a significant loss of dopaminergic neurons and accumulation of α-synuclein aggregates in the SN, marked reduction of dopaminergic terminals and dopamine content in the striatum, as well as increased expression of divalent metal transporter 1 and iron staining in SN and striatum (Baiguera et al., 2012). Furthermore, we described that the SN and striatum of 18-month-old c-rel^−/−^ mice displayed a significant increase in the area of CD11b-positive microglia (Baiguera et al., 2012). More recently, we found that c-rel^−/−^ mice displayed progressive accumulation of pathological α-synuclein in SN, as well as striatal loss of dopamine transporter (DAT) as early as 12 months of age, when they did not show either motor impairment or loss of dopaminergic neurons in the SN yet (Lanzillotta et al., 2015; Parrella et al., submitted). In this premotor phase (2–13 months of age), c-rel^−/−^ showed non-motor symptoms typical of PD, such as hyposmia and gastrointestinal dysfunctions (Lanzillotta et al., 2015; Parrella et al., submitted).

In this study, we investigated whether there is a temporal correlation between inflammatory transcription and microglial/astrocyte activation with the development of the spontaneous PD-like pathology in c-rel^−/−^ mice. We evaluated markers of inflammation and microglial/macrophage activation, as well as signs of gliosis, in the premotor and motor stages of c-rel^−/−^ mice at 4, 13 and 18 months of age.

## Materials and Methods

### Experimental Animals

C57BL/6 mice carrying the c-Rel gene null mutation (c-rel^−/−^) were originally generated by inserting the neomycin cassette into the fifth exon of the *c-Rel* gene (Liou et al., 1999). The genotypes were confirmed by PCR analysis (Baiguera et al., 2012) and western blot, and both lines were continued by homozygous breeding. The c-rel^−/−^ and c-rel^+/+^ wild-type (wt) mice were housed in the animal facility of the Department of Molecular and Translational Medicine of the University of Brescia. Animals were maintained in standard cages under 12/12 h light/dark cycles with *ad libitum* access to food and water. Humidity and room temperature were maintained constant. This study was carried out in accordance with the recommendations of the Directive 2010/63/EU of the European Parliament and of the Council of 22 September 2010 on the protection of animals used for scientific purposes. The protocol was approved by the animal-welfare body of the University of Brescia. Male 4-, 13- and 18-month-old mice were used for this study.

### Real-Time Quantitative Reverse Transcription-Polymerase Chain Reaction (qRT-PCR)

Total RNA was purified from SN using the RNeasy Mini Kit for total RNA extractions (Qiagen). Total RNA (1 μg) was reverse transcribed using the Quantitect^®^ Reverse Transcription Kit (Qiagen) according to manufacture instructions. Retrotranscribed cDNA (1–5 μL) was amplified using iQ™ SYBR Green Supermix (Bio-Rad) and 10 μM optimized forward and reverse primers. PCR reaction was performed using the standard program in ViiA7™ Real-Time PCR System (Applied Biosystems). Each reaction was performed at least in triplicate. For standardization of quantification, beta-Actin *(Actb)* was used as housekeeping gene. Data were analyzed following the comparative Ct method. The following primers were used for real-time quantitative reverse transcription-polymerase chain reaction (qRT-PCR):

- *arginase 1 (Arg1)*: For GTGTACATTGGCTTGCGAGA; Rev AATCGGCCTTTTCTTCCTTC
- *Actb*: For GGCTCTTTTCCAGCCTTCCT; Rev ATGCCT GGGTACATGGTGGT
- *Fc gamma receptor III (Fcgr3)*: For TATCGGTGTCAAATGGAGCA; Rev GCACCT TAGCGTGATGGTTT
- *Il6*: For CCTACCCCAATTTCCAATGCT; Rev TATTTT CTGACCACAGTGAGGAAT
- *Il1b*: For GGCTTCAGGCAGGCAGTATC; Rev TAATGG GAACGTCACACACC
- *mannose receptor 1 (Mrc1)*: For AAGGTTCGGGATTGTGGAG; Rev AATCGGCCTTTTCTTCCTTC
- *triggering receptor expressed on myeloid cells 2 (Trem2)*: For CACTCTGAAGAACCTCCAAGC; Rev ATTCCTGGAGGTGCTGTGTT
- *chitinase-like 3 (Ym1)*: For GCCCACCAGGAAAGTACACA; Rev CACGGCACCTCCTAAATTGT

### Immunofluorescence

Mice were anesthetized with chloral hydrate (400 mg/kg intraperitoneally) and transcardially perfused with PBS (Sigma-Aldrich) and 4% (w/v) ice-cold paraformaldehyde (PFA). Brains were postfixed in 4% PFA at 4°C for 2 h, followed by cryoprotection in 30% sucrose in PBS until cut. Coronal slices (15 μm-thick) were cut to obtain serial sections of the SN (anterior–posterior 2.54–3.40 mm), using bregma-based coordinates (Paxinos and Franklin, 2012).

Immunofluorescence for the ionized calcium binding adapter molecule 1 (Iba1) and glial fibrillary acidic protein (GFAP) antibodies was initiated by performing antigen retrieval (Tris-EDTA Buffer pH 9, 90°C, 10 min), followed by permeabilization and blocking. Then, sections were incubated overnight at 4°C with the primary antibodies rabbit anti-Iba1 (1:500, Wako #019-19741) or mouse anti-GFAP (1:100, Sigma #G3893). The following day, slices were incubated for 1 h at room temperature with the secondary antibodies goat anti-rabbit Alexa Fluor 488 (1:1000, Jackson ImmunoResearch #111-545-144), for Iba1, and goat anti-mouse Alexa Fluor 488 (1:800, Jackson ImmunoResearch #115-546-071), for GFAP. Lastly, sections were incubated with Hoechst 33342 (1 mg/mL, Sigma-Aldrich) to stain cellular nuclei.

Double immunofluorescence staining for MRC1 and Iba1 was initiated by performing antigen retrieval, permeabilization and blocking, followed by an overnight incubation at 4°C with rat anti-mouse MRC1 (1:50, Bio-Rad #MCA2235). Sections were then incubated for 1 h at room temperature in biotinylated goat anti-rat (1:400, Vector laboratories #BA-9401) followed by streptavidin-Alexa Fluor 488 (1:1000, ThermoFisher Scientific #S11227). Then, the sections were incubated 2 h at 37°C in the second primary antibody rabbit anti-Iba1, goat anti-rabbit Alexa Fluor 488, and Hoechst 33342.

Double immunofluorescence staining for IL-1β and Iba1 was performed by incubating sections in goat anti-Iba1 (1:100, Novus Biologicals #NB 300-270) overnight at 4°C followed by donkey anti-goat Alexa Fluor 488 (1:500, Jackson ImmunoResearch #705-545-147). Slices were then incubated in the second primary antibody rabbit anti-IL-1β (1:50, Abcam #ab9722) overnight at 4°C, followed by biotinylated goat anti-rabbit (1:400, Vector laboratories #BA-1000), streptavidin-Alexa Fluor 488, and lastly Hoechst 33342. In order to check antibody specificity, control (blank) reactions with no primary antibodies were run in parallel.

### Microscopy

Immunofluorescence stainings were analyzed by an inverted light/epifluorescence microscope (Olympus IX50; Olympus), and pictures were captured with a digital camera (Olympus XC 30) and cellSens Software (Olympus) and analyzed in a blinded manner. Images were adjusted for brightness and contrast with Adobe Photoshop (Adobe system) software.

### Statistical Analysis

Statistical analysis was performed with the GraphPad Prism program (GraphPad Software). qRT-PCR data was analyzed by two-way analysis of variance (ANOVA) followed by Bonferroni *post hoc* test. Data are presented as mean ± SEM. *P* < 0.05 was considered as significant.

## Results

### Age-Related Alterations in the Expression of Inflammatory and Microglial/Macrophage Activation Markers in the Substantia Nigra of c-Rel Deficient Mice

We first evaluated the expression of general markers of inflammation and specific markers for the diverse microglial/macrophage activation states, M1 (pro-inflammatory) and M2 (anti-inflammatory, phagocytic) by qRT-PCR on SN RNA extracts of wt and c-rel^−/−^ mice at 4, 13 and 18 months. In particular, we analyzed the pro-inflammatory markers *Il1b* and *Il6*, that are widely expressed in the CNS, and *Fcgr3*/CD16, that is specifically expressed by M1-polarized microglia/macrophages. Moreover, we determined the expression of *Mrc1*, *Ym1*, *Arg1* and *Trem2*, specific markers for the M2 activation state of microglia/macrophages.

**Figure 1:**
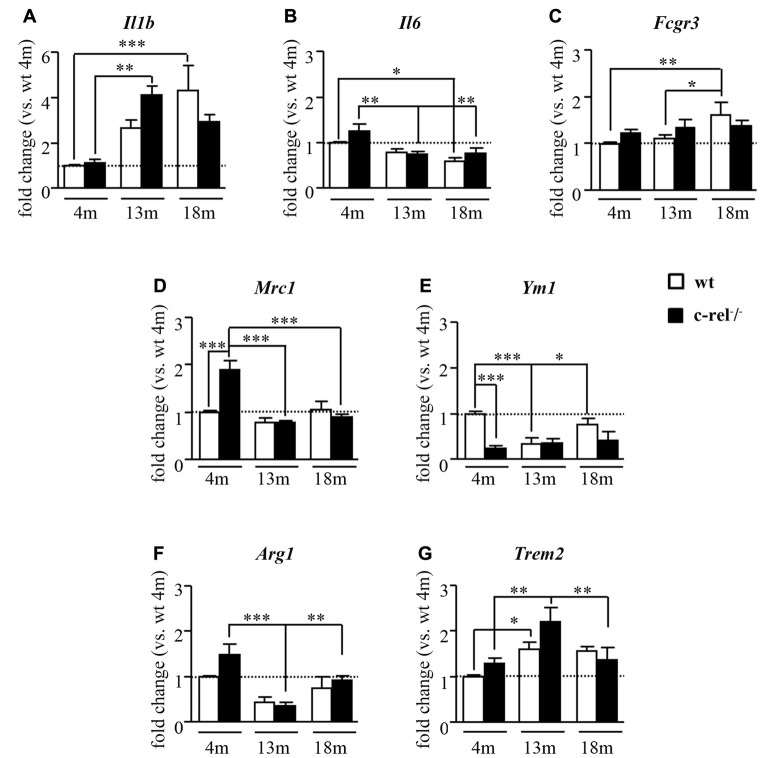
Inflammatory and microglial/macrophage mRNA expression profile in c-rel^−/−^ and wild-type (wt) mice. Real-time quantitative reverse transcription-polymerase chain reaction (qRT-PCR) analysis of pro-inflammatory cytokines, interleukin 1 beta (*Il1b*) and *interleukin 6* (*Il6*; **A,B**), and microglial/macrophage activation markers, *Fc gamma receptor III* (*Fcgr3*; **C**), for M1 phenotype; *mannose receptor 1* (*Mrc1*; **D**), *chitinase-like 3* (*Ym1*; **E**), *arginase 1 (Arg1)*; **(F)**, and *triggering receptor expressed on myeloid cells 2 (Trem2)*; **(G)**, for M2 state in substantia nigra (SN) of 4, 13 and 18 months of age mice (*n* = 4–6 animals per group). Four-month-old c-rel^−/−^ mice showed increased expression of M2c microglial marker *Mrc1* and *Arg1*, associated with marked decrease of *Ym1* transcription. At 13 months of age, c-Rel deficient mice displayed an upregulation in *Il1b* transcription compared to younger c-rel^−/−^ mice, while no differences in microglial M2 markers were found in c-rel^−/−^ mice compared to wt group. No biologically relevant variations in all the analyzed markers were evident in 18-month-old c-rel^−/−^ mice compared to wt. Data are presented as mean ± SEM. *P < 0.05, **p < 0.01, ***p < 0.001; two-way analysis of variance (ANOVA) followed by Bonferroni test for comparison vs. 4-month-old wt mice.

At 4 months of age, no differences were found in the mRNA expression of the pro-inflammatory markers *Il1b* and *Il6*, and M1 microglia/macrophage marker *Fcgr3* in the SN of c-rel^−/−^ mice when compared to wt (Figures 1A–C). At 13 months of age, a statistically significant increase of *Il1b* expression was detected only in c-rel^−/−^ mice (Figure 1A). This age-dependent increase in *Il1b* transcription was also detected in the wt mice at 18 months of age (Figure 1A). Interestingly, the levels of *Il1b* in 18-month-old c-rel^−/−^ mice were not significantly different from those observed at 4 months (Figure 1A). However, no biologically relevant, albeit statistically significant, differences in the expression of *Il6* or *Fcgr3* were observed between c-rel^−/−^ and wt mice at all the time points analyzed (Figures 1B,C). These analyses only showed an age-related decrease of *Il6*, occurring in both mouse lines, and a statistically significant increase of *Fcgr3* in 18-month-old wt mice (Figure 1C). On the other hand, the analysis of the expression of M2 markers in 4-month-old mice showed a significant increase of *Mrc1* and a trend to increase for *Arg1* transcription in c-rel^−/−^ mice when compared to wt, while *Ym1* resulted significantly downregulated (Figures 1D–F). No significant difference in *Trem2* was detected between c-rel^−/−^ and wt mice at this age (Figure 1G). This expression pattern seems to indicate a polarization of microglia/macrophages towards a particular M2 state named M2c, that is associated with tissue repair, as well as to debris and iron scavenging (David and Kroner, 2011), in 4-month-old c-rel^−/−^ mice. However, the M2c activation state was no longer detectable in 13-month-old c-rel^−/−^ mice. Indeed, at this age, *Mrc1* and *Arg1* transcripts resulted downregulated in c-rel^−/−^ mice when compared to 4-month-old mice, while *Ym1* expression remained unchanged (Figures 1D–F). On the other hand, we observed a significant increase in *Trem2* transcription in 13-month-old c-Rel deficient mice when compared to younger animals (Figure 1G). Though similar changes were found in wt mice (Figure 1G). Finally, we did not observe biologically relevant changes in the expression of all M2 markers between c-rel^−/−^ and wt mice at either 13 or 18 months of age, thus suggesting a limited involvement of the M2 polarization of microglia/macrophages in the SN of older c-rel^−/−^ mice.

These data support the occurrence of M2c-like pattern of expression in the SN of c-rel^−/−^ mice at 4 months of age, when they do not display any sign of degeneration in this area yet. It is followed by a mild inflammatory state characterized by *Ilb* expression in 13-month-old c-rel^−/−^ mice, when they develop a loss of DAT immunostaining in the striatum (Lanzillotta et al., 2015).

### Immunohistochemical Characterization of Mild Inflammatory Profile in the Substantia Nigra of c-Rel Deficient Mice

**Figure 2:**
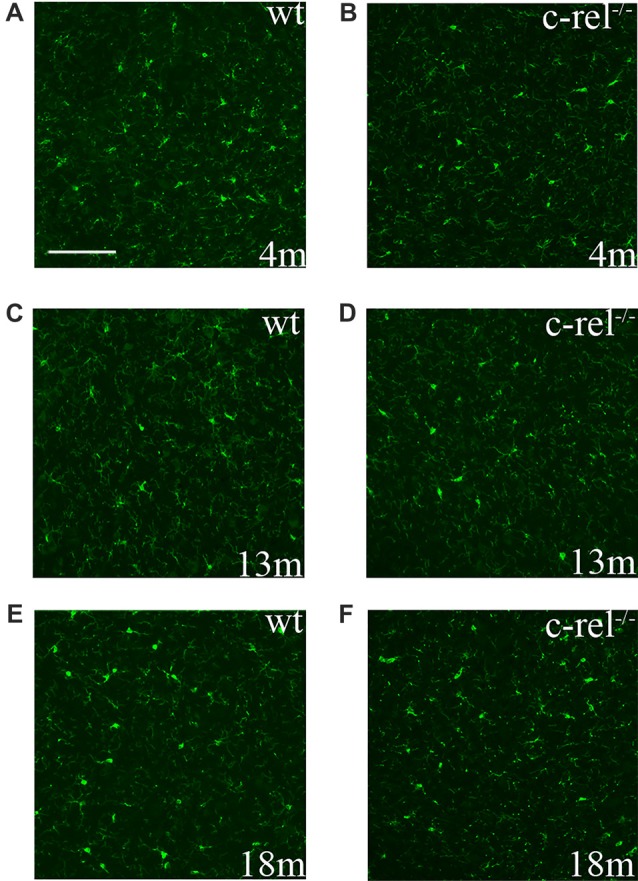
Ionized calcium binding adapter molecule 1 (Iba1) immunoreactivity in c-rel^−/−^ and wt mice. Pictures of SN sections from 4- **(A,B)**, 13- **(C,D)** and 18-month-old animals **(E,F)** illustrating Iba1 immunostaining. No evident changes in microglial activation were found in c-rel^−/−^ mice **(B,D,F)** compared to age-matched wt **(A,C,E)**. Immunofluorescence images are representative of five sections from each animal (*n* = 3 animals per group). Abbreviations: SN, substantia nigra. Scale bar: in **(A)** = 100 μm for **(A–F)**.

To assess microglial activation in the premotor and motor phases of PD-like pathology, we analyzed the immunoreactivity for Iba1, a constitutive marker of microglia and macrophages, in the SN of 4-, 13- and 18-month-old c-rel^−/−^ mice and age-matched wt animals. Iba1 was expressed similarly, both in distribution and morphology, in c-rel^−/−^ and wt mice, at all the considered ages (Figure 2). These data appeared contrasting to our previous findings showing an increased immunoreactivity for CD11b in the SN of 18-month-old c-rel^−/−^ mice (Baiguera et al., 2012).

**Figure 3:**
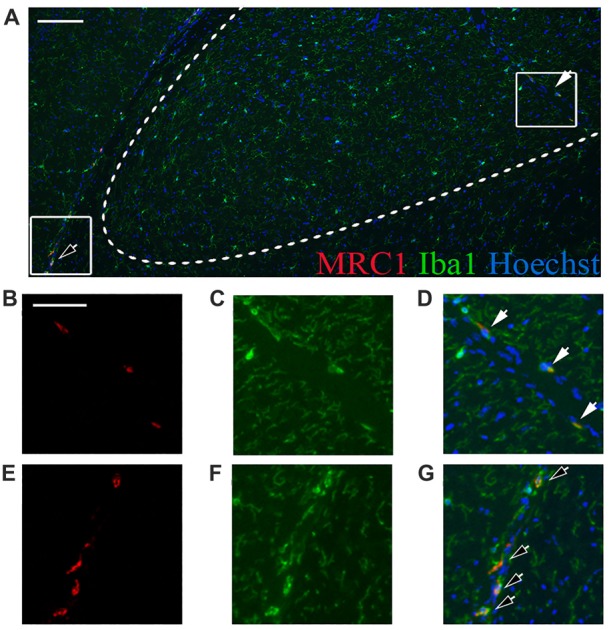
Non-parenchymal macrophages are immunoreactive to MRC1. **(A)** MRC1 (red) and Iba1 (green) double-labeled cells are localized at CNS interfaces (closed and open arrows) and not in the SN parenchyma (delimited by the dashed line) in 4-month-old c-rel^−/−^ mice. **(B–D)** Higher magnification of MRC1-Iba1 double-labeled perivascular macrophages (closed arrow) and Iba1-positive microglia in the SN. **(E,F)** Higher magnification of MRC1-Iba1 double-labeled meningeal macrophages (open arrows), and Iba1-positive microglia, outside the SN. Upper image **(A)** is representative of five sections from each animal (*n* = 3). Abbreviations: CNS, central nervous system; SN, substantia nigra. Scale bars: in **(A)** = 100 μm; in **(B)** = 50 μm for **(B–G)**.

To identify the origin of the increased *Mrc1* transcription in the SN of 4-month-old c-rel^−/−^ mice, double immunofluorescence staining with MRC1 and Iba1 was performed in brain sections of these animals. MRC1 immunoreactivity was not detected in Iba1-positive parenchymal microglia, but instead in Iba1-positive cells located at CNS interfaces (Figure 3). These non-ramified cells were identified as non-parenchymal or CNS macrophages, namely perivascular or meningeal, depending on their localization and morphology (Goldmann et al., 2016). Considering that the aim of the present work was to study parenchymal microglia in the SN, we chose not to proceed with the analysis of these few macrophages limited to CNS borders, though it would definitely be interesting to focus our attention on these cells in a future study.

**Figure 4:**
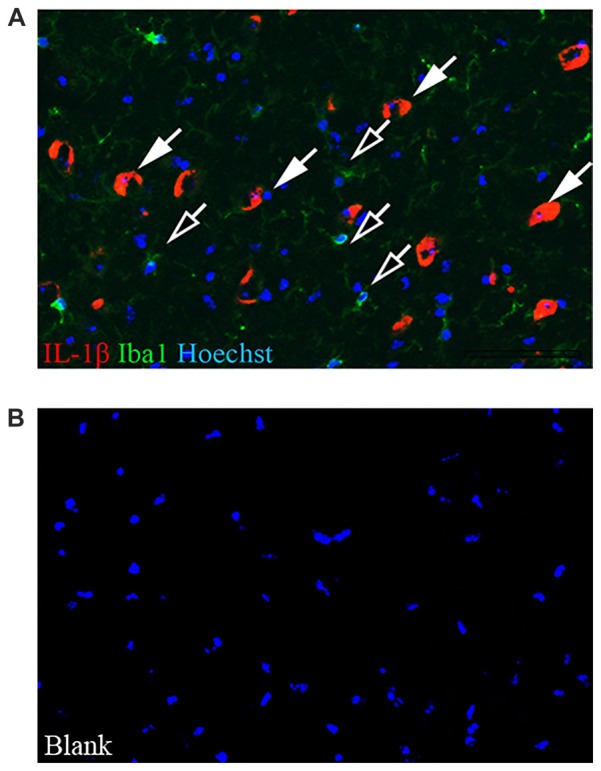
IL-1β localization in the SN. **(A)** Representative image of IL-1β (red) and Iba1 (green) immunofluorescence with Hoechst (blue) staining in the SN of 13-month-old c-rel^−/−^ mice. Closed arrows highlight IL-1β-positive cells. Open arrows highlight Iba1-positive cells. The image is representative of five sections from each animal (*n* = 3). **(B)** Blank control. Abbreviations: SN, substantia nigra. Scale bar: in **(A)** = 100 μm for **(A,B)**.

To establish the source of the increased *Il1b* mRNA in the SN of c-rel^−/−^ mice at 13 months of age, SN sections from these mice were immunolabeled for IL-1β and Iba1. IL-1β expression was mostly found in cells negative for Iba1 in 13-month-old c-rel^−/−^ mice (Figure 4). Considering their morphology, we speculate these IL-1β-positive Iba1-negative cells are neurons.

**Figure 5:**
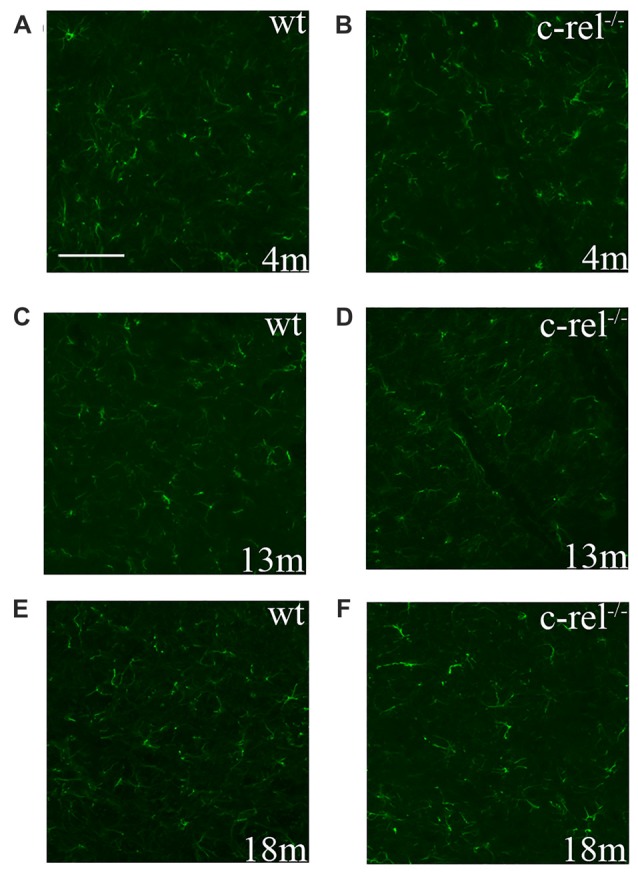
GFAP immunoreactivity in c-rel^−/−^ and wt mice. Pictures of SN sections from 4- **(A,B)**, 13- **(C,D)** and 18-month-old **(E,F)** animals illustrating GFAP astroglial immunostaining. No evident changes in astroglial activation were found in c-rel^−/−^ mice **(B,D,F)** compared to age-matched wt **(A,C,E)**. Immunofluorescence images are representative of five sections from each animal (*n* = 3 animals per group). Abbreviations: GFAP, glial fibrillary acidic protein; SN, substantia nigra. Scale bar: in **(A)** = 100 μm for **(A–F)**.

The immunoreactivity for GFAP, an astrocyte marker, was performed to analyze astrocyte activation in the SN of c-rel^−/−^ mice. In line with our earlier results (Baiguera et al., 2012), no signs of astrogliosis were detected in SN sections of 18-month-old c-rel^−/−^ mice. Likewise, no increased reactivity to GFAP was identified in 4- or 13-month-old c-rel^−/−^ mice, when compared to wt animals (Figure 5).

## Discussion

The present study shows that merely a mild inflammatory process, lacking a pronounced activation of microglia and astrocytes, anticipates SN degeneration in c-rel^−/−^ mouse, a novel animal model of late-onset parkinsonism (Baiguera et al., 2012).

Although several animal models of PD have been established in the last decades, none of them showed to develop both the parkinsonian motor and non-motor symptoms (Cebrián et al., 2015). Furthermore, most models display rapid dopaminergic neuron degeneration, not replicating the slow, aging-dependent neuronal loss characteristic of the sporadic human disease (Tieu, 2011). Recently, we have observed that, between 2 and 13 months of age, c-rel^−/−^ mice manifest olfactory deficits and gut dysfunctions, which are accompanied by progressive α-synuclein accumulation in the SN and reduction of DAT immunoreactivity in the striatum (Parrella et al., submitted). These alterations, that are reminiscent of prodromal PD, anticipate the onset of the motor disturbances and dopaminergic neuronal loss in the SN that become evident at 18 months (Baiguera et al., 2012). The aim of the present study was to investigate whether inflammation and glial activation may be associated with or anticipate the onset of PD-like neuropathological alterations reported in the brain of c-rel^−/−^ mice.

The analysis of inflammation-related transcripts in the SN of 4-month-old c-rel^−/−^ mice revealed increased expression of *Mrc1* and *Arg1* with respect to wt mice. The transcription of these factors was associated with a decreased *Ym1* expression, supporting the presence of the M2c-type microglial/macrophages associated with iron scavenging (David and Kroner, 2011) in the SN of c-rel^−/−^ mice. The M2c microglial/macrophage polarization appeared to be transient as it dropped down at 13 and 18 months of age. Although a role for M2c-type microglia/macrophages in the onset and progression of the PD-like disease has yet to be established, our findings suggest that M2c “sentinel cells” in the SN of c-rel^−/−^ mice are indeed reacting to harmful signals already present in brain milieu of young animals. On the contrary the loss of this M2c-phenotype might contribute to the accumulation of iron and likely α-synuclein in the SN of older c-rel^−/−^ mice (Baiguera et al., 2012).

The double labeling of MRC1 and Iba1 in the SN of these mice allowed us to identify the MRC1-Iba1-positive cells as non-parenchymal or CNS macrophages, rather than microglia. This is in agreement with recently published data demonstrating that the marker MRC1 is specific for CNS macrophages, but not microglia, in mice (Goldmann et al., 2016) and humans (Melief et al., 2012). It suggests that the observed changes in the expression of *Mrc1* might reflect macrophage rather than microglial alterations.

Notably, at 13 months of age, the reduction in the expression of M2c-specific markers was accompanied by a significant increase of *Il1b*, but not *Il6* or *Fcgr3* transcription, which is suggestive of a mild inflammatory profile in the c-rel^−/−^ brain. In wt mice, a similar increase of *Il1b* expression became evident only at 18 months of age. As an augment of IL-1β is recognized to be a feature of brain aging (Maher et al., 2004), the anticipation of *Il1b* expression in c-rel^−/−^ mice supports that c-Rel deficiency accelerates the aging and neurodegeneration of the SN (Baiguera et al., 2012; Lanzillotta et al., 2015). In addition, we found that immunoreactivity for IL-1β was mostly localized within Iba1-negative cells displaying a neuronal morphology in the SN of c-rel^−/−^ mice. However, we cannot exclude that other cell types, such as microglia, might contribute to the higher level of *Il1b* transcripts detected by qRT-PCR. This is in line with previous findings showing transcription but no transduction of IL-1β in peripheral blood mononuclear cells, as well as in the SN of a PD mouse model (Schindler et al., 1990a,b; Depino et al., 2003).

Anyhow, we could not detect differences in the morphology of Iba1-positive microglia/macrophages in the SN of c-rel^−/−^ mice, at all the considered ages, though we previously described the presence of activated CD11b-positive cells in the SN of 18-month-old c-rel^−/−^ mice (Baiguera et al., 2012). This discrepancy may be ascribed to the use of different microglial/macrophage markers in the two studies. Indeed, several authors reported that CD11b and Iba1 can lead to different staining patterns (Korzhevskii and Kirik, 2016; Lee et al., 2016; Scholtzova et al., 2017), which depends on the microglial/macrophage activation state (Ji et al., 2007). In particular, CD11b, although being an unspecific marker for microglia and macrophages, as it is also expressed in monocytes and granulocytes (Korzhevskii and Kirik, 2016), detects preferentially the activated microglial/macrophage state. This is related to the fact that resting microglia display low expression for membrane receptors, including CD11b, which become upregulated upon activation (Kreutzberg, 1996). On the other hand, Iba1 is a specific microglial/macrophage marker, but not especially sensitive to the activated form (Liaury et al., 2012; Korzhevskii and Kirik, 2016). Previous evidence by Liaury et al. showed increases in immunoreactivity for CD11b without significant difference in Iba1 in the hippocampal dentate gyrus of Gunn rats, an animal model of schizophrenia (Liaury et al., 2012). We can assume that microglia may adopt a minor “intermediate” state of activation detectable only by immunolabeling CD11b in c-rel^−/−^ mice. The Iba1-labeled microglia, by depicting microglial morphology at all the activation states, is a realistic representation of the mild inflammatory condition present in c-rel^−/−^ mice. The blunt inflammatory response is also supported by the GFAP staining for astrocytes in SN of c-rel^−/−^ mice. As previously reported (Baiguera et al., 2012), c-rel^−/−^ mice showed no signs of astrogliosis, accordingly to findings in PD patients (Mirza et al., 2000; Song et al., 2009; Tong et al., 2015). The role of astrocytes in PD pathology remains controversial (Hirsch and Hunot, 2009). Moreover, microglial activation does not necessarily lead to the release of proinflammatory cytokines (Depino et al., 2003). As mentioned above, it has been found that peripheral blood mononuclear cells, that are able to transcribe but not translate *Il1b*, upon receiving a secondary inflammatory stimulus, respond excessively by releasing disproportionate quantities of inflammatory cytokines, a phenomenon that is known as cell priming (Schindler et al., 1990a,b). Therefore, we hypothesize that this “intermediate” microglial activation state in 18-month-old c-rel^−/−^ mice could belong to “primed” microglia. Interestingly, neurodegenerative diseases and normal aging are responsible for microglial priming or sensitization (Norden and Godbout, 2013). In a model of accelerated aging, primed microglia showed an exaggerated response to peripheral lipopolysaccharide injection (Raj et al., 2014). Since PD patients frequently suffer from infections, being even one of the main causes of death (Beyer et al., 2001), it cannot be excluded that peripheral infections trigger an excessive brain inflammatory response at the symptomatic motor stage of the disease. Diverse factors, such as nutrition (Seidl et al., 2014; Agim and Cannon, 2015) and physical exercise (LaHue et al., 2016), that have been assumed to influence the progression of PD, could very well contribute to modulate the neuroinflammatory process (Spencer et al., 2012; Orr et al., 2013). If we consider that c-rel^−/−^ mice exhibit increased susceptibility to brain aging, it would be remarkable confirming the presence and role of primed microglia in this animal model, by evaluating its reactivity upon a peripheral inflammatory stimulus in further investigations. Moreover, our findings do not clarify the origin of a putative microglial “priming” and whether c-rel^−/−^ mice display an impaired neuronal resilience at 12 months of age, as suggested by the increased *Il1b* expression in SN and loss of DAT in the striatum (Lanzillotta et al., 2015).

Definitely, whether and how inflammation contributes to PD pathology is still debated. Post-mortem analysis of PD brains has shown reactive microglia in the SN of patients at the end stage of the disease (McGeer et al., 1988; Imamura et al., 2003). The assessment of microglial activation *in vivo* at earlier stages of PD can be achieved by positron emission tomography (PET) imaging of radioligand binding to mitochondrial translocator protein 18 kDa (TSPO), a molecule expressed during microglial activation (Banati et al., 1997). However, genetic polymorphisms in TSPO cause an elevated variability in binding affinity, which is the main limitation of this technique (Owen et al., 2012; Yoder et al., 2013). In two studies that have controlled for TSPO rs6791 polymorphism, no differences were seen in [18F]-FEPPA binding in PD patients compared to controls (Koshimori et al., 2015; Ghadery et al., 2017). Thus, whilst microglial activation seems to be a pathological hallmark of end-stage PD, small-scale studies on the activation of microglia in patients at initial phases of the disease suggest there is no association of microgliosis with early disease progression. These findings are also supported by recent investigations showing no difference in CSF cytokine content in patients with mild PD (Shi et al., 2011; Lindqvist et al., 2013). By rising some concerns about the role of inflammation as a trigger of the disease, this scenario deserves confirmation in large-scale studies.

In conclusion, our findings hint that severe inflammation, and particularly microglial pro-inflammatory activation, is not a key hallmark of PD-like phenotype in c-Rel deficient mice. So far, we have analyzed microglial activation in mice up to 18 months of age, when dopaminergic neuronal loss in the SN is about 40% (Baiguera et al., 2012). The moderate amount of neuronal degeneration leads us to speculate that, at this age, c-rel^−/−^ mice are modeling a mild PD. Thus, we cannot exclude that c-rel^−/−^ mice may develop strong microgliosis at older ages, similar to what is found in post mortem brains of late-stage PD subjects (McGeer et al., 1988; Imamura et al., 2003). These findings own potentially relevant implications for understanding the role of neuroinflammation in PD.

## Author Contributions

VP, MM and MP conceived and designed the experiments; VP, MM, EP and MB performed the experiments; VP, MM and LF analyzed the data; PT and PFS contributed to results interpretation; VP, MM, AB and MP wrote the article.

## Conflict of Interest Statement

The authors declare that the research was conducted in the absence of any commercial or financial relationships that could be construed as a potential conflict of interest.

## Abbreviations

Arg1: arginase 1
CNS: central nervous system
CSF: cerebrospinal fluid
DAT: dopamine transporter
Fcgr3: Fc gamma receptor III, also known as CD16
GFAP: glial fibrillary acidic protein
Iba1: ionized calcium binding adapter molecule 1
Il6: interleukin 6
Il1b: interleukin 1 beta
Mrc1: mannose receptor 1
PD: Parkinson’s disease
qRT-PCR: real-time quantitative reverse transcription-polymerase chain reaction
SN: substantia nigra
Trem2: triggering receptor expressed on myeloid cells 2
Ym1: chitinase-like 3.
